# Early Biodistribution and Persistence of a Protective Live Attenuated SIV Vaccine Elicits Localised Innate Responses in Multiple Lymphoid Tissues

**DOI:** 10.1371/journal.pone.0104390

**Published:** 2014-08-27

**Authors:** Deborah Ferguson, Giada Mattiuzzo, Claire Ham, Richard Stebbings, Bo Li, Nicola J. Rose, Edward T. Mee, Deborah Smith, Mark Page, Martin P. Cranage, Neil Almond, Greg J. Towers, Neil J. Berry

**Affiliations:** 1 Division of Virology, NIBSC, South Mimms, Hertfordshire, United Kingdom; 2 Division of Biotherapeutics, NIBSC, South Mimms, Hertfordshire, United Kingdom; 3 Institute of Infection and Immunity, St. George's, University of London, London, United Kingdom; 4 MRC Centre of Medical Molecular Biology, Division of Infection and Immunity, UCL, London, United Kingdom; Beth Israel Deaconess Medical Center, Harvard Medical School, United States of America

## Abstract

Vaccination of Mauritian cynomolgus macaques with the attenuated *nef*-truncated C8 variant of SIVmac251/32H (SIVmacC8) induces early, potent protection against pathogenic, heterologous challenge before the maturation of cognate immunity. To identify processes that contribute to early protection in this model the pathogenesis, anatomical distribution and viral vaccine kinetics were determined in relation to localised innate responses triggered by vaccination. The early biodistribution of SIVmacC8 was defined by rapid, widespread dissemination amongst multiple lymphoid tissues, detectable after 3 days. Cell-associated viral RNA dynamics identified mesenteric lymph nodes (MLN) and spleen, as well as the gut mucosae, as early major contributors of systemic virus burden. Rapid, localised infection was populated by discrete foci of persisting virus-infected cells. Localised productive infection triggered a broad innate response, with type-1 interferon sensitive IRF-7, STAT-1, TRIM5α and ApoBEC3G genes all upregulated during the acute phase but induction did not prevent viral persistence. Profound changes in vaccine-induced cell-surface markers of immune activation were detected on macrophages, B-cells and dendritic cells (DC-SIGN, S-100, CD40, CD11c, CD123 and CD86). Notably, high DC-SIGN and S100 staining for follicular and interdigitating DCs respectively, in MLN and spleen were detected by 3 days, persisting 20 weeks post-vaccination. Although not formally evaluated, the early biodistribution of SIVmacC8 simultaneously targets multiple lymphoid tissues to induce strong innate immune responses coincident at the same sites critical for early protection from wild-type viruses. HIV vaccines which stimulate appropriate innate, as well as adaptive responses, akin to those generated by live attenuated SIV vaccines, may prove the most efficacious.

## Introduction

Development of an effective vaccine against HIV/AIDS remains an important global public health target. Modest but short-lived efficacy has been obtained in a recent human vaccine trial using an avipox vector prime and Envelope subunit boost [1]. However, to make a major impact on the pandemic, improved vaccine strategies that elicit durable, broadly effective, potent protection will be required. Live attenuated vaccination (LAV) with simian immunodeficiency virus (SIV) in non-human primates has provided proof of concept of highly efficacious vaccine protection [2], [3], demonstrating durable, potent resistance against detectable superinfection with wild-type SIV challenge by multiple routes and with diverse virus strains [4]–[20]. Identification of the mechanism(s) of protection has proven to be difficult, perhaps, at least in part, due to the focus on the measurement of responses in the peripheral circulation. Indeed, the largest study conducted to date has confirmed the absence of immune responses associated with vaccine protection in peripheral blood [11].

Moreover, potent vaccine-generated protection and resistance to superinfection *in vivo* may be generated shortly after vaccination with certain attenuated SIV strains [18], [19], [12], [7], [8], at a time when the cognate immune response is absent or immature in quantity and quality. Hence, we were interested to characterise the innate immune responses generated in the early, immediate post-vaccination phase. Whilst it is unclear whether a single mechanism mediates protection at early and late times after vaccination, the relative contributions of both the cognate adaptive and innate arms of the immune response are important to define, particularly since innate responses have been relatively understudied and will likely influence protective adaptive responses [21], [22]. A valuable feature of live attenuated vaccines is the ability to stimulate long-lived protective immunity. Moreover, in common with live vaccines in general, the degree of protection conferred by attenuated SIV is inversely proportional to the degree of viral attenuation [13], [23]. Despite the overall success of live attenuated vaccines, it is only recently that the immune parameters of this vaccine approach are being unravelled. Increasingly, the importance of cognate innate signalling in the conditioning of appropriate adaptive immune responses is being realised [24]. While safety concerns prevent the direct application of live lentivirus vaccine approaches in humans, defining the protective processes elicited by attenuated SIV may inform the design of novel approaches for a safe, durable and effective vaccine against HIV.

In our studies conducted in the Mauritian cynomolgus macaque (MCM, *Macaca fascicularis*) model with the attenuated *nef*-disrupted C8 variant of SIVmac251/32H (SIVmacC8), we have been able to demonstrate that protection against wild-type strains can be generated as early as 21 days after vaccination [17], [18], [7], [8], including against heterologous viral challenge [7]. As efficient vaccine replication *in vivo* appears to be a crucial prerequisite to long-term protection and early vaccine virus replication leads to changes in lymphocyte populations in gut-associated lymphoid tissue [25], we sought to understand the viral kinetics *in vivo* at multiple localised sites of infection and how localisation and dissemination of the SIVmacC8 virus influences innate responses associated with vaccination/infection. Mauritian CM provide an excellent model to examine this topic, representing a genetically well-characterised host with a limited host MHC composition due to founder population effects [26]–[30].

In a detailed early pathology study of the infecting time-course we describe how the SIVmacC8 vaccine virus is rapidly established in an early, widespread and persisting infection in multiple lymphoid tissues (LT), which is by no means confined to the gut mucosae. Concurrent with this localised infection of LT, we identify concomitant early stimulation of components of the innate immune system. We analysed the induction of a range of anti-viral factors including TRIM5α, ApoBEC3G (A3G), BST-2/tetherin, TRIM22 [31]–[39], the dendritic and myeloid restriction factor SAMHD1 [40]–[41] and mediators of type-1 interferon signalling STAT-1 and IRF-7. In parallel, cell surface expression changes of markers of cell lineages orchestrating innate immune responses including macrophages and dendritic cells (DC) [42]–[44] were induced as early as day 3, which persisted in the MLN and spleen coincident with persisting vaccine virus replication.

In determining the biodistribution of this live attenuated SIV we unravelled a strong induction of aspects of innate immunity which occur in tandem in multiple tissues in response to a localised and persisting vaccine virus. Persistence of this viral vaccine 3–20 weeks post-vaccination appears to drive a concomitant localised and persisting innate response, not confined to the gut mucosae. The implications of these findings in studies of early live attenuated SIV vaccine protection are discussed and pave the way for further detailed differential studies of viral vaccine protection.

## Results

### Distribution and kinetics of intracellular virus in lymphoid tissue

**Figure 1:**
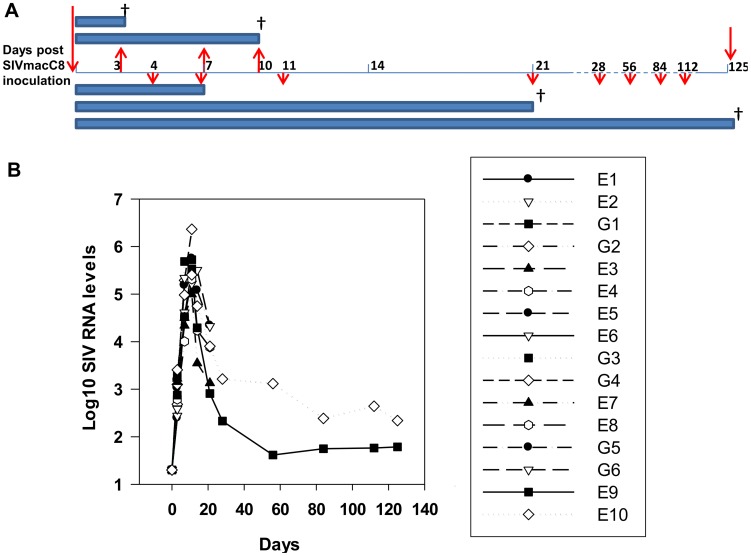
Study time-course and plasma vRNA levels. **A)** Schematic outline of experimental details. Pairs of juvenile MCM were serially sacrificed at days 0, 3, 7, 10, 21, 125 post intravenous vaccination with 0.5 ml of a 1/10 dilution of the 9/90 pool of SIVmacC8. Intermediate blood sampling times are indicated by red arrows. **B)** Plasma viral RNA levels determined by qRT-PCR at each time point for plasma samples collected during acute infection (days 3, 7, 10 and 21 p.i.) and the chronic phase (day 125). Macaques analysed *post-mortem* were (E1, E2, G1, G2, day 3; E3, E4, day 7; E5, E6, G3, G4, day 10; E7, E8, G5, G6, day 21 and E9, E10, day 125).

A total of 16 Mauritian cynomolgus macaques, for which the MHC haplotype had been determined (Figure S1), were inoculated intravenously with SIVmacC8 in an early pathology study of live attenuated SIV. Groups of animals were sequentially sacrificed at days 3, 7, 10 and 21 across the acute phase (Figure 1A). For comparison, two macaques were monitored into the chronic phase of infection and sacrificed at 125 days post infection (d.p.i.) for additional tissue analysis. Individual plasma viral RNA (vRNA) kinetics of each macaque indicated all were infected to comparable levels displaying highly reproducible vRNA kinetics between individuals (Figure 1B). Peripheral viral load peaked 10 days p.i. (4.5–5.5 log_10_ SIV RNA copies/ml); all vaccinates subsequently displayed a controlled profile of plasma vRNA kinetics, although macaques E9 and E10 differed slightly in their long-term control of plasma viraemia to 125 days p.i. (Figure 1B).

**Figure 2:**
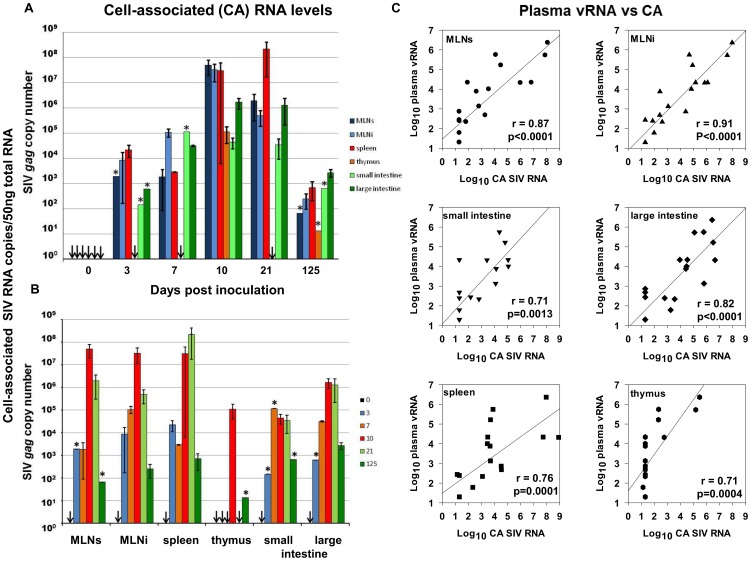
Rapid and widespread dissemination of SIVmacC8 to multiple lymphoid tissues during the acute phase of infection. **Panels A, B**. Cell-associated (CA)- RNA concentrations determined over the time-course in tissue-derived cells. 50 ng total RNA extracted from tissue-derived cells was processed in a one-step probe-based qRT-PCR. SIV *gag* copy number derived from a standard curve was normalised to 5×10^5^ copies of GAPDH. Histograms represent average values from 2 (time points 7 and 125 days p.i.) or 4 (0, 3, 10 and 21 days p.i.) animals, performed in duplicate in at least two independent experiments (±SEM). SIV *gag* copy number was plotted against time in days post-inoculation (**panel A**), or grouped by tissue (**panel B**). Data were obtained for superior and inferior mesenteric lymph nodes (MLNs and MLNi, respectively), spleen, thymus and small and large intestine. * denotes results obtained from a single animal. Downward arrows indicate no signal detected – all tissues at day 0, (naïve, baseline) or in the thymus at days 3, 7 and 21. **Panel C** shows the relationship between plasma vRNA levels and CA-RNA levels for each tissue. Log_10_ values are represented as a scatter plot and the correlation between the two variables determined by linear regression analysis with r^2^ values are shown.

**Figure 3:**
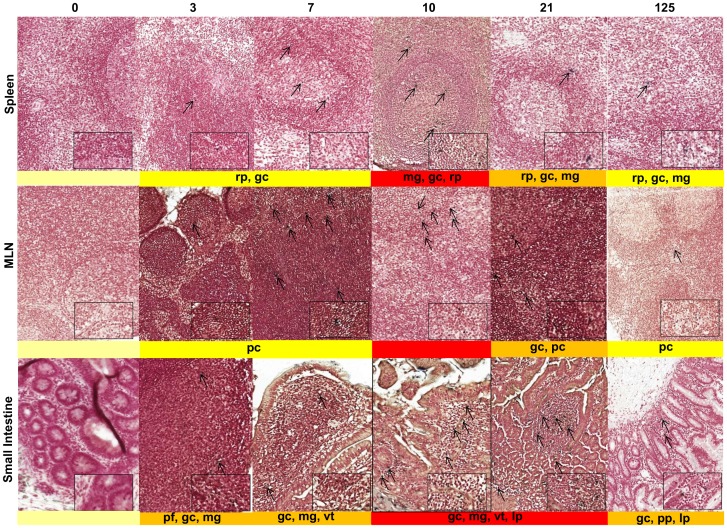
SIVmacC8 establishes persisting foci in infection in multiple lymphoid tissues. Galleries of representative fields of *in-situ* hybridisation of sections of spleen, MLN and small intestine in MCM inoculated with SIVmacC8 and sacrificed at 0 (naïve) 3, 7, 10, 21 and 125 days post infection (d.p.i). Heatmap staining representing the overall frequency of foci of virus-infected cells in multiple fields is shown beneath individual panels. Location of foci of virus-infected cells are represented as indicated: pf – primary follicles, pp - peyers patches; pc – paracortex, rp – red pulp, gc – germinal centre, mg – follicular marginal/mantle zone, ms – medullary sinuses, lp – lamina propria, vt – villi tips within each main tissue type and indicated by arrows. Samples from naive, uninfected macaques are shown as controls. Magnification was ×20, main image; ×80 inset. *In situ* hybridisation for combined SIV *gag/env/nef* (g/e/n) messenger RNA conform to the same classification of staining intensity as IHC for comparative purposes: pale yellow (no staining, −); yellow (very low, +); dark yellow (medium, ++), red (high, +++), corresponding to the mean number of positive cells/mm^2^: + (0.5–6.8), ++ (6.9–13.8) and +++ (>13.8) cells/mm^2^ also represented in Table S1.

The quantity and distribution of SIVmacC8 cell-associated RNA (CA-RNA) in multiple lymphoid tissues, compared by real-time quantitative PCR (qRT-PCR; Figures 2A, B) and *in situ* hybridisation (ISH, Figure 3, Table S1), revealed intracellular viral kinetics of early infection to be defined by a rapid and simultaneous dissemination of SIVmacC8 to multiple lymphoid tissues. Tissue-specific differences were compared with baseline ISH data generated from a total of 15 naïve Mauritian cynomolgus macaques; the distribution and number of viral foci for each tissue were related to these. Early dissemination of SIVmacC8, characterised by CA-RNA detected 3 days p.i. in organised lymphoid tissues of superior and inferior mesenteric lymph nodes (MLN), spleen, small and large intestines was reflected by the presence of virus-infected foci detected by ISH in MLN, spleen and small intestine (SI). Typically, the concentration of CA-RNA determined by qRT-PCR and frequency of virus-infected cells as shown in Figures 2 and 3 increased over time in all tissues, peaking 7–21 days p.i. SIVmacC8 thus established an active, persisting infection in all tissues sustained beyond the immediate cessation of the primary viraemic phase. This was corroborated by both ISH and qRT-PCR, although some subtle tissue-specific differences were identified (Figures 2 and 3, Table S1).

### Contributions of different lymphoid tissues to the total viral burden

All tissues sampled supported actively replicating virus, not limited to any one anatomical site. Each compartment contributed to the total systemic virus burden reflected in plasma vRNA load in peripheral blood during acute infection. Comparisons of concentrations of CA-RNA levels for the 6 lymphoid regions sampled (inferior and superior MLN, large and small intestine, spleen and thymus) and the total plasma vRNA at each sampling point (Figure 2C) identified a positive correlation in all tissues. Inferior and superior MLN CA-RNA concentrations particularly showed a high degree of correlation with plasma virus, in terms of production and cessation of virus during the acute phase. The lower correlation between splenic CA-RNA and plasma vRNA can be accounted for by the delayed peak in intracellular RNA to day 21 (Figure 2A, B), where concentrations in this tissue remained high when virus levels in the blood have begun to wane (days 10–21 p.i.). Hence, the intracellular virus concentrations in the MLN most closely correlated with the dynamics of the primary plasma viraemia.

Overall viral spread and expansion reflected in these increased CA-RNA concentrations were accompanied by a greater density of viral foci identified at day 10 in spleen and MLN, although there was not always a direct correlation between the number of viral foci and concentrations of SIV RNA in different tissues. In the MLN, detection of viral RNA by ISH was maximal at day 10 in the germinal centres, follicular marginal and mantle zones. In the small intestine, foci of infection were observed in primary follicles, follicular and mantle zones and germinal centres at day 3. By day 7, virus had spread to the villi tips and at the peak of primary virus replication (days 10–21) had invaded the lamina propria. Virus persistence was reflected in overall distribution in the small intestine at day 125 with virus detected in the Peyer's patches, germinal centres and lamina propria (Figure 3). Tracking of virus through the small intestine, MLN and spleen indicated that by day 21 p.i., all three tissues were characterised by persisting virus-infected cells that expressed viral RNA.

Cell-associated viral DNA (vDNA) levels showed a similar picture although, as expected, CA-RNA loads were a more sensitive measure of intracellular virus (Figure S2).

### Induction of restriction factors in multiple lymphoid tissues

**Figure 4:**
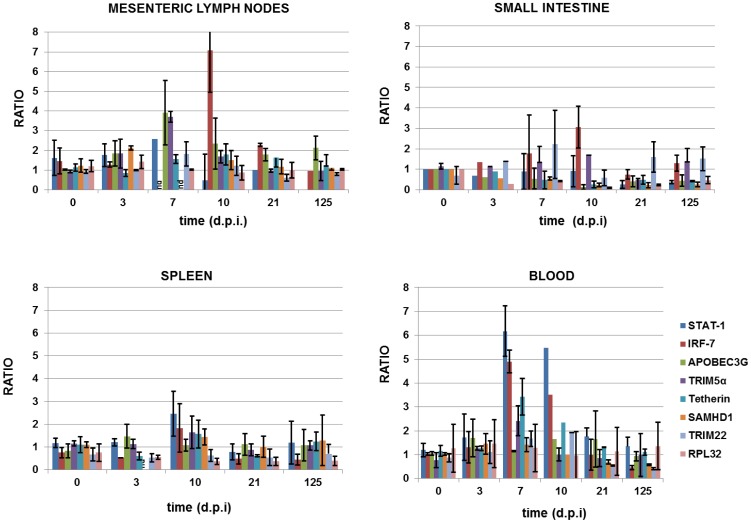
Induction of interferon-dependent genes in tissue-derived cells. Expression levels of interferon signalling genes (IRF-7, STAT-1), restriction factors (TRIM5α, APOBEC3G, tetherin, SAMHD1, TRIM-22), and ribosomal protein L32 (RPL32) were represented as fold difference from levels in naïve macaques, after normalisation using housekeeping gene GAPDH. Data are plotted for mesenteric lymph nodes, small intestine, spleen and blood across the time course from naïve (day 0) to 3, 7, 10, 21 and 125 d.p.i. Histograms represent average values from 2–4 animals ± SEM. The equivalent of 50 ng of reverse transcribed total RNA was used in a SYBR-green based qPCR.

The impact of localised virus replication on induction of known restriction factors (RF) and interferon-stimulated genes (ISG) was assessed using the total RNA extracted in Figure 2. Relative gene expression levels were determined by qRT-PCR for TRIM5α, ApoBEC3G, BST-2/tetherin, TRIM-22, SAMHD-1, STAT1 and IRF7 comparing levels in naïve macaques normalised to GAPDH (Figure 4). ISGs IRF-7 and STAT-1 were induced 7–10 d.p.i., particularly in blood, coincident with peak virus production. Not all RF genes were upregulated in all tissues. ApoBEC3G and TRIM5α mRNAs were upregulated during acute SIVmacC8 infection, though not in all tissues. This could not be attributed to the presence of different TRIM5α genotypes since only three TRIM5α genotypes are present amongst these Mauritian animals with no TRIM-cyp variants and the qPCR used has been validated for these sequences [29], [30]. Tetherin mRNA was poorly induced in tissues, although tetherin mRNA levels peaked in PBMC. As expected, there was no IFN-dependent upregulation of SAMHD1 [40], [41], [45] nor, more surprisingly, TRIM22 mRNA. Hence, SIVmacC8 induced transient, sporadic induction of selective antiretroviral RFs in at least three LT. It is difficult to determine the impact of RF/ISG induction due to differences in viral kinetics and host responses among a limited number of animals. Low numbers in each group also precluded a formal statistical analysis of the relationship between RF/ISG induction and tissue-specific viral load, although there was a clear trend towards upregulation coincident with high levels of CA-RNA in different LT. Moreover, it is clear that RF activation elicited during peak infection, did not prevent persistence of SIVmacC8 and its widespread distribution among multiple LT.

### SIVmacC8 activates a broad spectrum of markers of innate immune function

**Figure 5:**
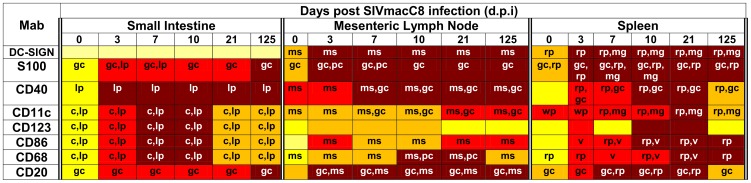
Heatmap showing cell surface staining for dendritic cells, macrophages and B cells. Staining intensities are as indicated shown for dendritic cell markers (DC-SIGN, S100, CD40, CD11c, CD123, CD86), macrophages (CD68) and B cells (CD20). Relative localisation of staining within each tissue are represented by: pc – paracortex, rp – red pulp, wp - white pulp, gc – germinal centre, mg – follicular marginal/mantle zone, ms – medullary sinuses, c – crypts, lp – lamina propria, within each main tissue type. Mab, monoclonal antibody. IHC gradings: pale yellow (no staining, −); yellow (very low, +); dark yellow (low, ++), red (medium, +++) and magenta (high, ++++).

**Figure 6:**
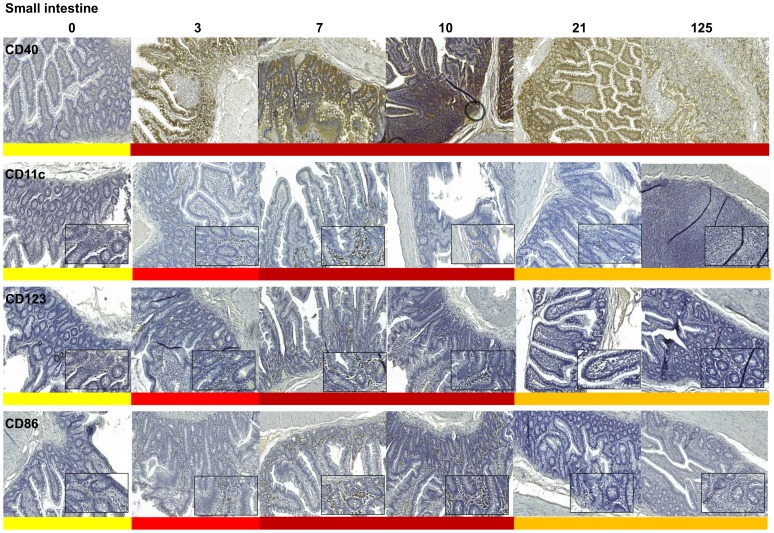
Expression of dendritic cell markers CD40, CD123, CD11c and CD86 in small intestine. Staining intensities are the same key as for Figure 5; ×10 magnification; inset, ×40, days post SIVmacC8 inoculation.

Further evidence for innate immune activation after vaccination was sought by detection of a range of cell surface activation markers expressed on DCs, macrophages and B-cells in the SI, MLN and spleen by immunohistochemistry (IHC, Figures 5 and 6, Table S2; Figures S3 and S4). Following vaccination with SIVmacC8 there was widespread innate immune activation measured by upregulation of all markers assessed by blinded scoring above baseline levels of naive, unvaccinated MCM. The kinetics of induction differed between tissues. In the small intestine, DC markers CD11c, (a type 1 transmembrane protein), CD123, (an IL-3 receptor also found on plasmacytoid dendritic cells (pDC)), CD86, (expressed on antigen presenting cells providing co-stimulatory signals for T-cell priming) and the macrophage marker CD68 all had very similar profiles exhibiting a marked upregulation in response to acute infection but returning to low levels 21–125 days p.i., mainly in the crypts and lamina propria (Figure 6). Upregulation of the CD123 marker suggests pDCs are recruited to the small intestine and transiently activated during acute infection. As pDCs are potent secretors of IFN-α, their transient induction is consistent with an acute response to virus infection in the gut that is not sustained in chronic infection and compatible with a burst of ISG induction (Figure 4).

Upregulation of granzyme B and perforin was observed 3–21 days p.i. in all three lymphoid tissues (Figure S5), reflecting the primary virus replication profile, before returning to baseline. Broad parameters of immune function including CD4, CD8 and CCR5 varied among tissues p.i. (Figure S5). Importantly, additional markers of DC activation, *eg* CD40, (a member of the TNFα superfamily) were induced to high levels 3–7 d.p.i. in the MLN and SI, but this upregulation persisted through to 125 d.p.i. CD40 expression varied in the spleen and was only expressed to high levels during acute infection. Differences in markers of innate immune function between local sites of infection suggest a complex innate immune response to vaccination, as might be expected.

### SIVmacC8 vaccination induces sustained DC-SIGN expression in MLN and spleen but not small intestine

**Figure 7:**
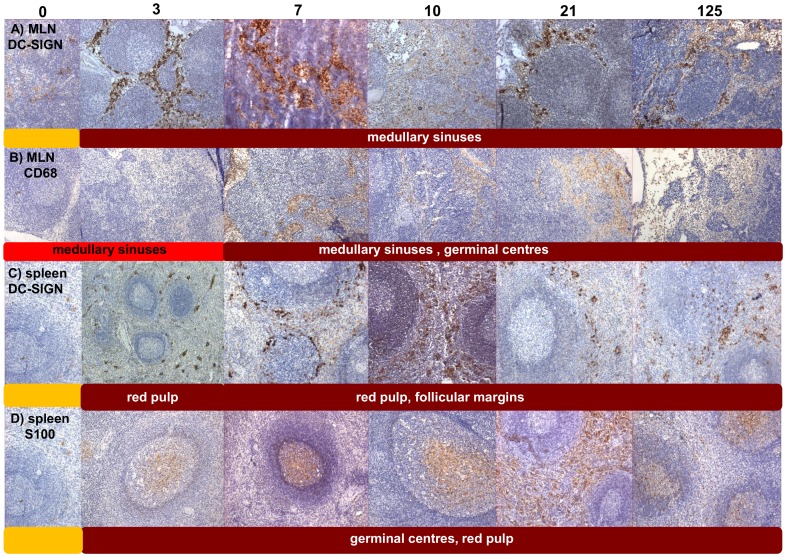
Rapid induction and persistence of DC-SIGN, S100 and CD68 post-vaccination in lymphoid tissue. Galleries of representative fields of **A** DC-SIGN and **B** CD68 staining in MLN at 3, 7, 10, 21, 125 d.p.i. **C** Representative fields of DC-SIGN signal tracking through the spleen 3–125 d.p.i. maximal from d3 in red pulp (rp) and marginal zones (mg) **D** Representative fields of splenic S100 levels illustrate similar staining intensity 3–125 d.p.i. predominantly in the germinal centres and red pulp. Staining intensities scoring were; pale yellow (no staining, −); yellow (very low, +); dark yellow (low, ++), red (medium, +++) and magenta (high, ++++).

IHC detection of different markers of innate immune function revealed that the spleen and MLN exhibited significant acute responses but importantly, also sustained upregulation of multiple markers persisting into chronic infection. DC-SIGN and S100 (markers of follicular and interdigitating DCs), CD40, CD11c, CD123, CD86, as well as CD68 and CD20 all exhibited high levels of expression at 21 d.p.i in spleen (Figure 5, Figure S3); however, only DC-SIGN, S100, CD86 and CD68 maintained persistent, high expression 125 d.p.i. Critically, we identified high levels of DC-SIGN, S100 and CD68 staining as early as 3 d.p.i., which persisted to chronic infection in MLN and spleen (Figures 5 and 7). Intense staining for DC-SIGN antigen was also found in the red pulp, follicular margins and mantle zones of the spleen but not in the SI. S100 staining was at intermediate levels 3–21 d.p.i. and maximal 125 d.p.i. in the SI. Images of comparative staining intensities for CD40, CD11c, CD123 and CD86 in spleen, MLN and SI are as shown in Figure 6 and Figures S4 and S5.

Overall, the data suggest that both cellular and cell-autonomous innate immune responses are activated at early time points after SIVmacC8 vaccination but persist only in selected tissues, notably MLN and spleen. Finally, the B cell marker CD20 was expressed at intermediate levels during acute infection but higher in the chronic phase in different tissues (Figure 5, Figure S6).

## Discussion

Identification of critical immune responses that might need to be elicited by an effective HIV vaccine represents a crucial milestone for logical vaccine development. Live attenuated SIV vaccination of macaques delivers potent protection that correlates with *in vivo* replication efficiency of the viral vaccine [13], [23]. Nonetheless, LAV strains characteristically exhibit low or undetectable steady-state plasma viraemia. Whereas, typically, virus replication is ongoing in lymphoid tissue [11]. Here, we sought to understand the anatomical niches, timing and distribution of the SIVmacC8 viral vaccine at lymphoid sites during the establishment of infection with a view to illuminating vaccine persistence and identifying local anti-viral responses as potential effectors of vaccine-mediated protection, focussing on innate responses. Moreover, the live attenuated SIV vaccine used in this study profoundly differs from strains used by other groups in that it confers protection at a time before maturation of cognate immunity; hence we sought to define the induction of these early responses.

Intravenous inoculation of live attenuated SIVmacC8 elicited rapid viral dissemination, reflected in widespread distribution of actively replicating virus-infected cells among multiple organs detectable 3–7 days p.i. Concordant data from intracellular SIV-specific qRT-PCR and *in situ* hybridisation performed on RNA purified from disaggregated lymphoid cells and formalin fixed paraffin embedded tissues respectively, revealed a dynamic picture of early virus replication not confined to the gut mucosae. While large numbers of susceptible CD4^+^ T cells reside in the small intestine, particularly the lamina propria [46], [47], with dynamic changes in lymphocyte subsets associated with an acute inflammatory response immediately post SIVmacC8 vaccination [25], the MLN and spleen were also identified as major sites of early vaccine virus replication. CA-RNA levels in MLN most closely paralleled plasma vRNA kinetics identifying it as a dominant source of systemic viraemia, in addition to the intestinal mucosae during acute infection [48]–[50]. Changes in location and quality of SIV infection in lymphoid tissues have also been reported in rhesus macaques (RM) infected with SIVmac239Δ*nef* [11] and in cynomolgus macaques infected with wild-type SIV [51].

We hypothesise that localised responses at these specific sites of virus replication *in vivo* are triggered by SIVmacC8 which may act either independently, or in concert with other facets of immunity, to protect these anatomical niches from incoming wild-type viruses. Persistence of antigen-expressing, virus-infected cells in target tissues that remain as peripheral viral loads fall supports this model. Indeed, by 21 days post-vaccination all tissues exhibited evidence of persisting infection. Although all tissues were targeted during primary infection and LAV persisted in multiple tissues, we sought to identify the early responses that might relate to vaccine protection, particularly measures of innate immune activation that associate with infected cells. In the Mauritian model of early SIVmacC8 protection, this may be particularly pertinent given our previous observations of early vaccine protection [7], [8], [18], [19].

In this unique longitudinal study of the early parameters of SIVmacC8 vaccination, the highly reproducible vaccine virus kinetics, well-characterised MHC background and limited TRIM5 genotype significantly mitigate the relative small study numbers (n = 2–4) at each time-point. Moreover, the relatively conserved genetics of these animals provided a remarkably stable host background upon which to conduct such studies, given the more outbred nature of other macaque cohorts, which can confound study outcomes due to, amongst other things, the impact of different TRIM5 genotypes [33], [34]. Taken together, however, it was possible to identify a clearly discernible pattern of responses to infection/vaccination during the critical acute phase where some responses were clearly transient in nature, while others were more persistent.

Induction of interferon stimulated genes including restriction factors and key members of the interferon response pathways (IRF-7 and STAT-1) suggested a type 1 interferon response to LAV infection, consistent with previous observations of both SIV [4] and HIV-1 [52]. However, fold changes in total mRNA induction were weak, suggesting induction in only a small percentage of cells. It is highly unlikely that any induction of TRIM5α in these Mauritian-derived macaques would be biased by polymorphism, given the limited diversity of TRIM5 genotypes in this species [29], [30]. We therefore assume that TRIM5α did not present a confounding variable in this study. We used IHC techniques seeking to specifically identify induction of innate markers within tissues. As the precise distribution of virus infection varied between different lymphoid compartments, methods were used to detect changes in immune cell subsets to identify responses that might co-localise with the virus.

Although unable to differentiate between upregulation of cell-surface expression on cells resident in tissues and migrating cells our data suggest profound immune changes at a local level. Markers for dendritic cell family members (DC-SIGN, S-100 and CD40) exhibited expression patterns that, like virus-infected cells, increase over 3–7 days p.i., and most importantly, were present in tissues where virus-infected cells persisted (Figures 5–7). This was particularly apparent in the spleen where elevated expression persists in the presence of ongoing intracellular viral replication, and was at its highest level 21 days post vaccination. Viral persistence and the high splenic virus turnover, suggested by viral load measurement (Figure 2), is compatible with the early protection generated by SIVmacC8 as well as with vaccine recrudescence in protected vaccinates challenged with wild-type SIV [8], [7]. Changes in CD4, CD8, CCR5, perforin and Granzyme B expression were not associated with development of host control of viral infection (Figure S5). It is notable that DC-SIGN and CD68 were upregulated from day 3 p.i. to the highest levels measured suggestive of a highly sensitive, effective induction of innate immune responses prior to development of any functionally relevant adaptive response. We hypothesise that this continued stimulation of myeloid lineages, particularly DCs and macrophages in critical tissues, may be an important component of the early protection generated by live attenuated SIV vaccines with dendritic and macrophage cell lineages in key anatomical niches playing a central role in this process. However, inclusion of a challenge component would be required to formally determine any association between these observations and the protection observed in this model.

Studies of other live virus vaccines (*eg* yellow fever) have shown strong induction of innate immune signals, including Toll-like receptors and DCs, which stimulate a robust adaptive response [53]–[55]. Yet the specific role of adaptive immunity in the spectrum of LAV mediated protection is far from clear. Indeed, there is a conundrum to identify immune responses that a LAV elicits that is highly effective in preventing infection with homologous wild-type virus, yet incapable of eliminating the vaccine virus [56]. Colonisation of immunologically privileged CD4^+^ T follicular helper (T_FH_) cells in the germinal centres of lymphoid tissue provides one possibility [11], [57]. In addition, trapping and accumulation of viral antigen in follicular dendritic cells along with recently produced virus provides a mechanism for continued immune stimulation. High SIVmacC8 RNA concentrations in the spleen are compatible with SIV infection of follicular dendritic cells presenting *gag* antigen and CD4^+^ cell frequencies in LT which occur with increased activation of T_FH_ cells, but not increased CCR5 expression [58]. Specific analysis of T_FH_ and their role as an early reservoir of virus infection would help clarify their contribution to early vaccine protection.

Despite rapid stimulation of CD20-expressing B cells compatible with expansion of the plasmablast during acute infection and co-resident virus in these cells, there appears to be little evidence, however, that adaptive humoral responses are directly linked to host protection [6]. Re-stimulation of memory B-cells during superinfection frequently boosts antibody titres which appear to represent a marker of retroviral infection, rather than a protective correlate. However, strong induction of innate immunity will likely influence development and maturation of antibodies, even if at low affinity, which may impact on protective processes. Absence of adaptive immune correlates of early protection is further highlighted by CD8^+^ T cell ablation across a 35 day period encompassing primary infection which neither prevents control of the primary viraemia, nor the ability to resist superinfection [19]. However, it remains possible that mechanistically distinct processes may be operating at early and later times particularly given demonstration of maturation of protection [3], [9], [11], [13], where induction of a strong innate response during the early period may also influence differential processes that occur at later times.

Depletion of target cells during the early primary phase of virus replication and dissemination provides one possible alternative explanation for different protective mechanisms at late and early times. That multiple lymphoid tissues support vaccine virus replication soon after intravenous administration and localised responses are detected in key secondary organs of MLN and spleen, the ability to resist challenge via different routes (*eg* mucosal sites) would be interesting to clarify. Viruses entering via different sites also track through secondary lymphoid tissues, so pre-existing vaccine-mediated responses in the spleen and MLN would likely also be protective in this scenario.

It is also important to recognise that Vpx-encoding viruses belonging to the HIV-2/SIVsm/SIVmac lineage, such as those used in this study, may interact quite differently with the innate immune system than Vpx-negative viruses such as HIV-1. Vpx antagonises the restriction factor SAMHD1 and may allow replication in myeloid lineage cells that are not permissive to HIV-1 *in vivo* [40], [41], [59]. Thus, SIVmac-based LAV may elicit a range of persisting immunological changes, including those which impact on macrophages and dendritic cell function that may not directly translate to HIV-1 infection in humans. However, dissecting the changes that occur in myeloid and lymphoid cell populations, as recently described with a conditionally replication-competent vaccine [60], will likely be crucial to understanding protective mechanisms and identifying novel approaches to HIV vaccination.

In conclusion, appropriate induction of components of the innate immune system, in tandem with adaptive responses, may be a requirement of highly efficacious HIV vaccines.

## Materials and Methods

### Study design and sample collection

A total of 16 juvenile, weaned Mauritian cynomolgus macaques were inoculated with 10 MID_50_ of the SIVmacC8 9/90 pool [61] and pairs sacrificed at days 3, 7 10, 21, and 125 post inoculation as previously described [25]. Blood and tissues were collected as shown in Figure 1A, consisting of inferior (i) and superior (s) mesenteric lymph nodes (MLN), spleen, small and large intestines.

### Ethics statement

Non-human primates were used in strict accordance with UK Home Office guidelines, under a licence granted by the Secretary of State for the Home Office which approved the work described. Animal work at NIBSC is governed by the Animals (Scientific Procedures) Act 1986 which complies with the EC Directive 86/609 and performed under licence (PPL 80/1952) granted only after review of all procedures in the licence by the NIBSC local Ethical Review Process (ERP). All study macaques were purpose bred and group-housed for the entire duration of the study, with daily feeding and access to water *ad libitum*. Given the limited availability of suitable macaques, age, sex and weight matching was not possible, nor central to the study outcome. Regular modifications to the housing area were made by husbandry staff including introduction of novel structures (*eg* swings and perching stations) and foodstuffs in novel manners to encourage foraging for food, to further enrich the study environment. The environmental temperature was 15–24°C, appropriate for macaques and rooms subject to a 12 hour day/night cycle of lighting. Animals were acclimatised to their environment and deemed to be healthy by the named veterinary surgeon prior to inclusion on the study.

All animals were sedated prior to bleeding or virus inoculation by venepuncture. Frequent checks were made by staff and any unexpected change in behaviour by individuals on study followed up, including seeking of veterinary advice where necessary. Regular blood samples were obtained to assess haematological parameters in blood that might provide evidence of incipient disease and veterinary advice sought when persisting abnormalities detected. The study was terminated and all animals killed humanely by administering an overdose of ketamine anaesthetic prior to development of overt symptomatic disease. All efforts were made to minimise animal suffering, including provision of a high standard of housing quarters and monitoring of animal health and well-being and the absence of procedures not essential to the study.

### Host genetics

The MHC haplotype and TRIM5 genotype was determined for all study Mauritian cynomolgus macaques (*Macaca fascicularis*) as previously reported [28]–[30].

### Quantification of SIV RNA levels in plasma

Plasma viral RNA levels were quantified by a real-time qRT-PCR method as previously described [8]. The sensitivity of detection was 50 SIV RNA copies/ml plasma.

### Cell-associated RNA isolation and quantitative RT-PCR

Total RNA from tissue-derived cells was extracted using an RNeasy kit (Qiagen) and subjected to on-column DNAase treatment according to the manufacturer's instructions. SIV *gag* was quantified by adaptation of a one-step quantitative RT-PCR as previously described [8] with 50 ng of total RNA input. SIV *gag* copies were quantified using a standard curve obtained by serial dilution of the plasmid SIV3+ [62]. Data were expressed as copies per 50 ng RNA, normalised to a GAPDH house-keeping gene. Sensitivity of detection was 20 SIV RNA copies/50 ng input RNA. For quantification of restriction factor transcripts, 500 ng of total RNA was subjected to reverse transcription using AccuScript High Fidelity reverse transcription kit (Agilent), in a final volume of 20 µL, using primers described in Table S3. One tenth of the reaction was processed in a quantitative PCR (qPCR) using Quantitect SYBR-Green PCR kit (Qiagen) with 0.4 µM of each primer. Each reaction was run in duplicate using the Mx3005P (Agilent), thermocycling conditions were: 50°C, 2 min; 95°C, 15 min; 40 cycles of 95°C, 15 sec, 55°C, 1 min and 60°C, 1 min. Specificity of qPCR products was assessed by dissociation curves and quantified using the 2^−ΔCt^ method, and the housekeeping gene GAPDH as normaliser. For selected genes quantification was verified using standard curves obtained by serial dilution of plasmids encoding GAPDH, RPL32, TRIM5α, SAMHD1 or tetherin. All data were analysed using MxPro software (Agilent).

### *In situ* hybridisation

Representative sections of LT and SI were collected *post-mortem*, fixed in 10% formal saline and embedded in paraffin wax using standard histological procedures. Four micron sections were cut and mounted on poly-L lysine coated slides. Prior to treatment sections were de-waxed in xylene and re-hydrated via graded ethanol∶water solutions. *In situ* hybridisation for detection of SIV RNA transcripts was performed with digoxigenin (dig; Roche, Lewes, UK), labelled single stranded DNA probes [51] in a cocktail containing three sense/antisense probes to SIV transcripts (*gag, env, nef*) using a BondMax automated staining machine, utilising the Research Mode option for protocol design and execution (Leica Microsystems USA) as previously described [63]. Quantification of ISH positive cells collected *post-mortem* for spleen (Spl), mesenteric lymph nodes (MLN) and small intestine (S.I.) was performed by manually counting all positive cells within 10 random fields of view (×10 lens and ×10 eye-piece magnification; equivalent to an area of 2.2 mm^2^). The mean number of positive cells/mm^2^ was expressed using a grading system of: + (0.5–6.8), ++ (6.9–13.8) and +++ (>13.8) cells/mm^3^ as previously reported [7]. Baseline values, −, (0) were established from a total of 15 additional uninfected Mauritian cynomolgus macaques.

### Immunohistochemical analyses and antibody staining

Immunohistochemical analyses were performed as previously described [64]. Unmasking of antigens to allow binding of the antibody was undertaken by the optimal technique for each combination of antibody and antigen. Sections were heated at full power (800W microwave) for five minutes fully immersed in Vector unmasking solution (Vector Laboratories, Peterborough, UK) previously heated to 96°C for immunolabelling of CD4 (H370, Santa Cruz Biotechnology Inc, California, USA), CD20 (L26), S100 (DAKO, Ely, Cambridgeshire, UK), DC-SIGN (120507, R+D Systems Inc, Minneapolis, USA), CD123 (6H6, eBioscience, San Diego, USA) and CD40 (11E9), CD11c (5D11) (Novacastra, Leica Microsystems, USA), or 10 mM citrate buffer (pH 6), KK13 (SIV gp120), [65]. Sections were incubated in 50 µg ml^−1^ proteinase K (Roche, Lewes, UK) in PBS pH 7.4 for 15 minutes at 37°C prior to immunolabelling with CD8 (C8/144B), CD68 (KP1) (DAKO, Ely, Cambridgeshire, UK), CCR5 (3A9), CD86 (fun-1) (Pharmingen, Oxford, UK), granzyme B (11F1, Novacastra, Leica Microsystems, USA) and perforin (5B10, Vector Laboratories, Peterborough, UK). Data were generated by assessing staining levels across the whole tissue section (at least 5 fields ×10 lens/10× eyepiece magnification) on blinded samples and tabulated results were generated for the mean score of all animals within that group. Representative fields were selected for presentation in figures and colour coded relating to the level of intensity of staining. IHC gradings were: pale yellow (no staining, −); yellow (very low, +); dark yellow (low, ++), red (medium, +++) and magenta (high, ++++). Staining levels for individual antibodies were compared to baseline values for naïve macaques.

## Supporting Information

Figure S1 **MHC profiles of 16 Mauritian cynomolgus macaques in the time-course study.** Each major haplotype (M1–M6) is represented by a different colour bar as depicted, or as a recombinant (rec), for MHC class IA, class IB and class II.
(TIFF)

Figure S2 **Quantitative DNA levels of multiple tissues post SIVmacC8 vaccination.** Copies of SIV DNA are expressed per 100,000 MNCs, error bars shown for multiple macaques sampled at each time-point. Abbreviations: MLNs and MLNi, superior and inferior mesenteric lymph node; PLN, peripheral lymph node. Downward arrows indicate no signal.
(TIFF)

Figure S3 **Expression of dendritic cell markers CD40, CD123, CD11c and CD86 in spleen.** Staining intensities are the same key as for Figure 5; ×10 magnification.
(TIFF)

Figure S4 **Expression of dendritic cell markers CD40, CD123, CD11c and CD86 in mesenteric lymph nodes (MLN).** Staining intensities are the same key as for Figure 5; ×10 magnification; inset, ×40, days post SIVmacC8 inoculation.
(TIFF)

Figure S5 **Heatmap showing staining for cell surface markers for viral env, CD4, CD8, CCR5, granzyme B and perforin.** Monoclonal antibody staining intensities are shown for SIV *env*, CD4, CD8, CCR5, granzyme B and perforin. Relative localisation of staining within each tissue is shown: represented by the following key: pp; peyers patches; pc – paracortex, rp – red pulp, 1′ – primary follicle, wp - white pulp, gc – germinal centre, gch, -germinal centre haze, mg – follicular marginal/mantle zone, ms – medullary sinuses, c – crypts, lp – lamina propria, vt – villi tips within each main tissue type. Mab, monoclonal antibody.
(TIFF)

Figure S6 **Staining intensities for CD20 B cell marker in spleen, MLN and small intestine during the time course of SIVmacC8 infection.** Staining intensities as depicted in Figure 5, days post SIVmacC8 inoculation.
(TIFF)

Table S1 **Quantification of ISH positive cells collected** ***post-mortem*** **for spleen, mesenteric lymph nodes (MLN) and small intestine following SIVmacC8 vaccination (days post-inoculation).**
(DOCX)

Table S2 **Comparative immunohistochemistry staining intensities for different markers for dendritic cells, macrophages and B cells.**
(DOCX)

Table S3 **Primer sequences for qPCR for multiple gene targets.**
(DOCX)

Checklist S1 **ARRIVE Checklist.**
(DOC)
