# Supplementary figures and images for: Early Biodistribution and Persistence of a Protective Live Attenuated SIV Vaccine Elicits Localised Innate Responses in Multiple Lymphoid Tissues
Source: PLoS One. 2014 Aug 27;9(8):e104390. doi: 10.1371/journal.pone.0104390 (PMC4146474; doi:10.1371/journal.pone.0104390)

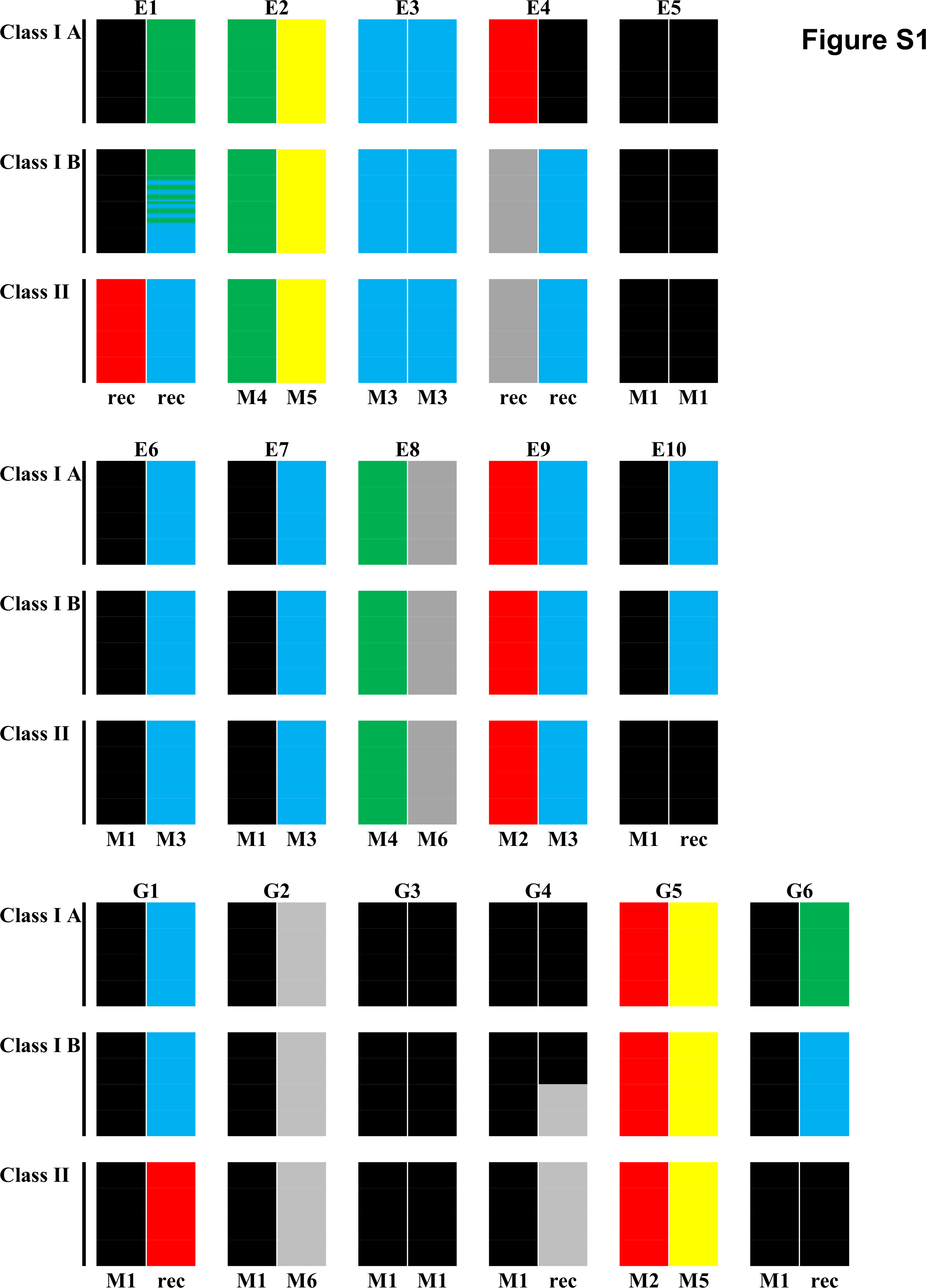

Supplement: Figure S1 — MHC profiles of 16 Mauritian cynomolgus macaques in the time-course study. Each major haplotype (M1–M6) is represented by a different colour bar as depicted, or as a recombinant (rec), for MHC class IA, class IB and class II. (TIFF) [file pone.0104390.s001.tiff]

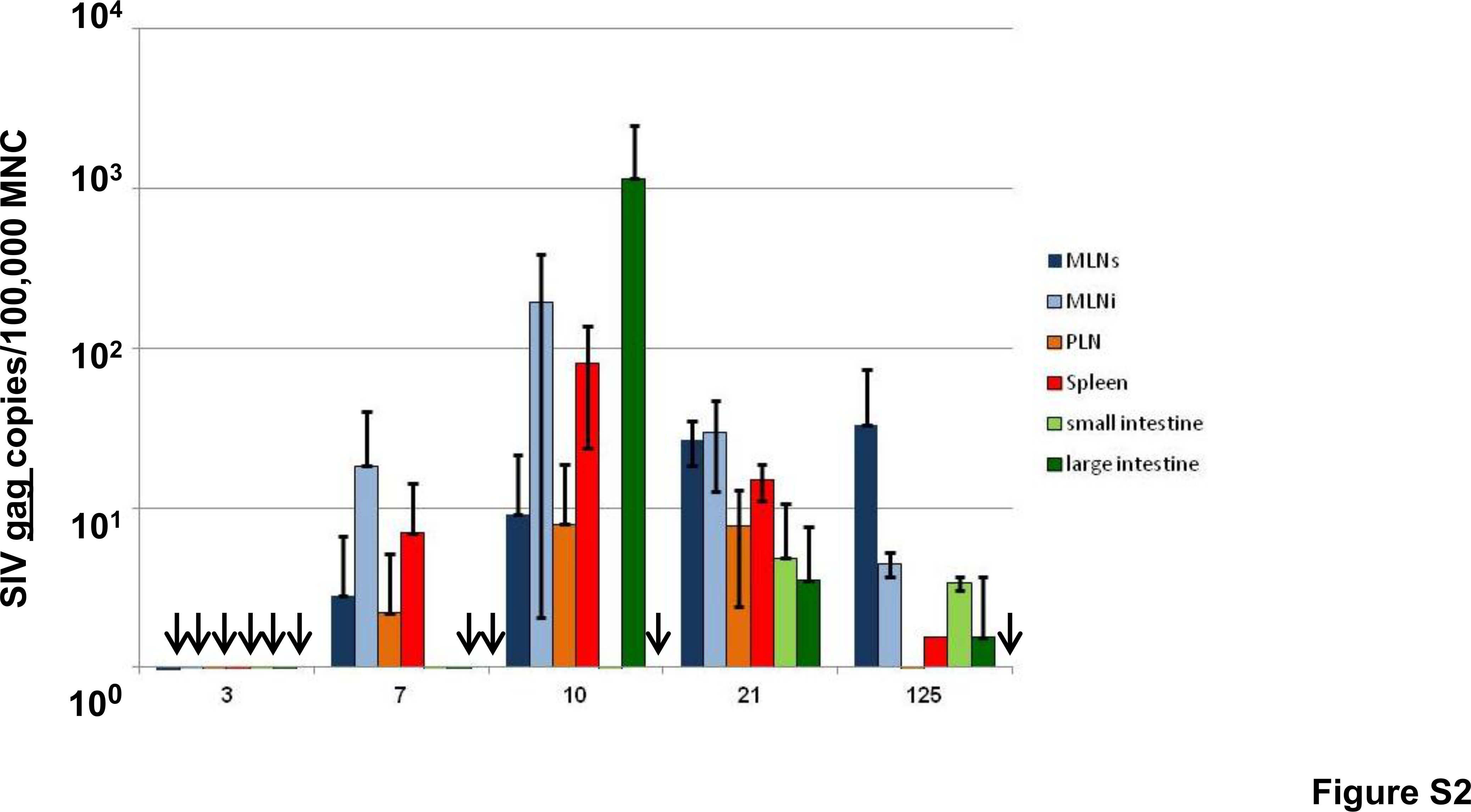

Supplement: Figure S2 — Quantitative DNA levels of multiple tissues post SIVmacC8 vaccination. Copies of SIV DNA are expressed per 100,000 MNCs, error bars shown for multiple macaques sampled at each time-point. Abbreviations: MLNs and MLNi, superior and inferior mesenteric lymph node; PLN, peripheral lymph node. Downward arrows indicate no signal. (TIFF) [file pone.0104390.s002.tiff]

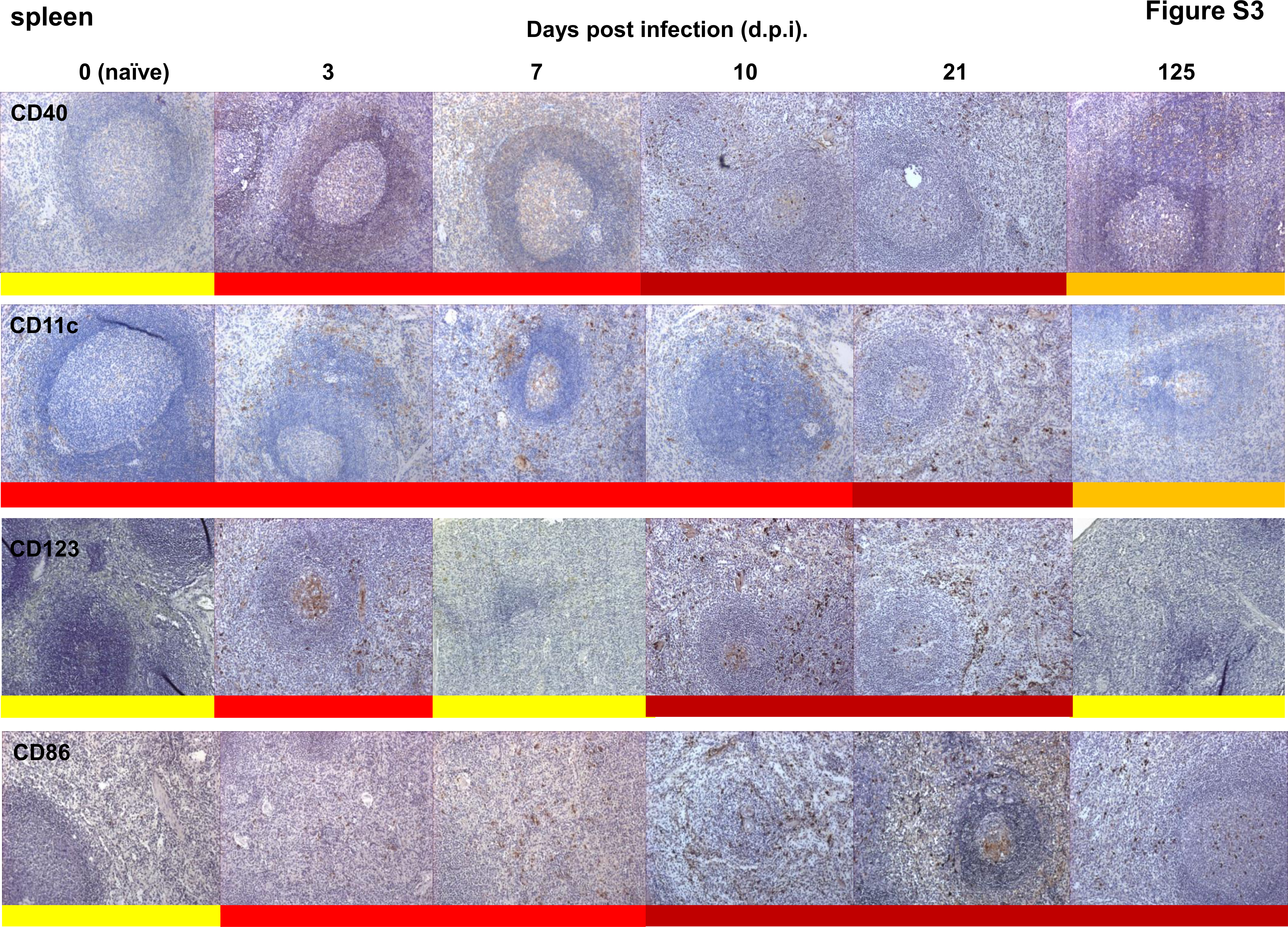

Supplement: Figure S3 — Expression of dendritic cell markers CD40, CD123, CD11c and CD86 in spleen. Staining intensities are the same key as for Figure 5; ×10 magnification. (TIFF) [file pone.0104390.s003.tiff]

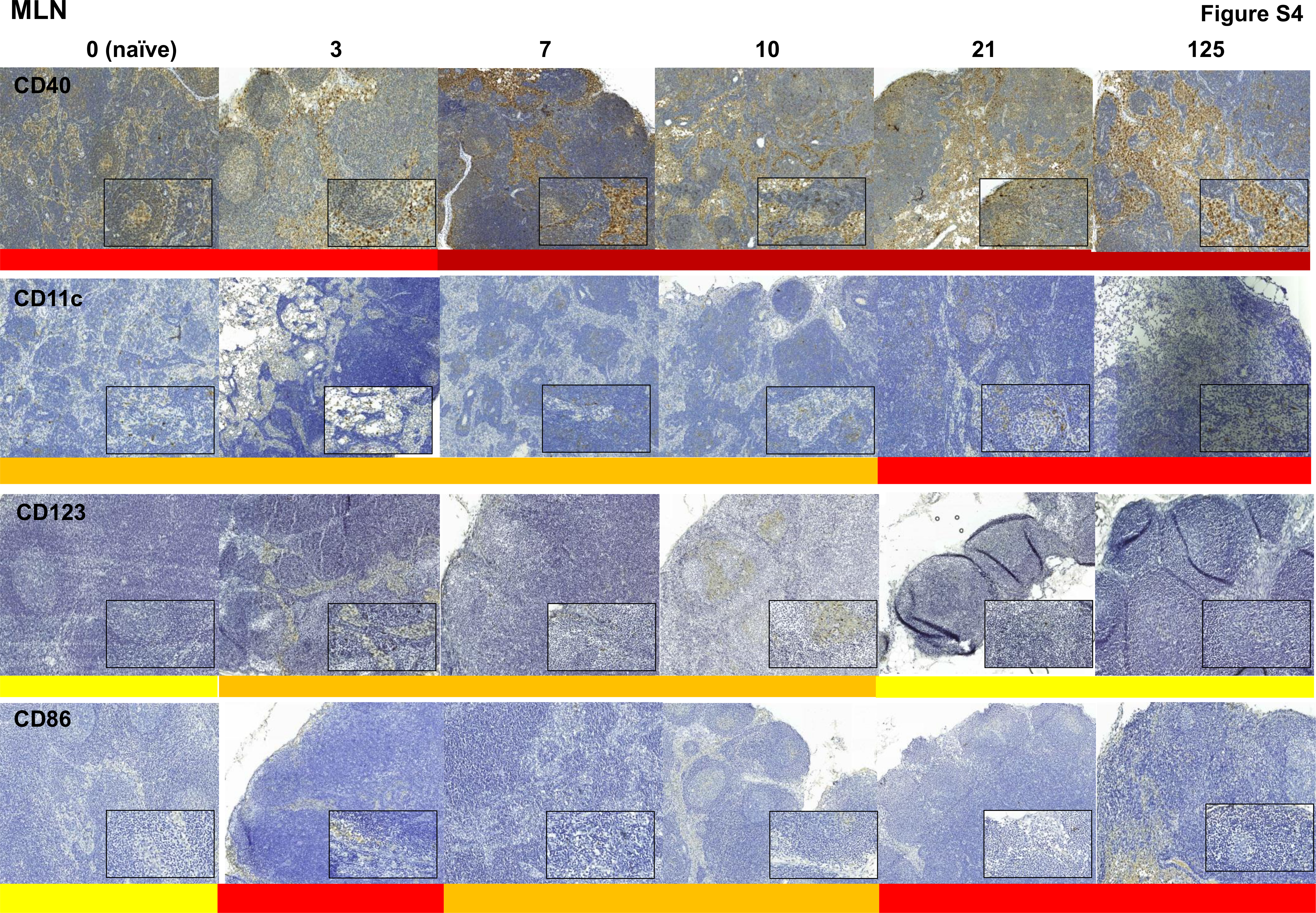

Supplement: Figure S4 — Expression of dendritic cell markers CD40, CD123, CD11c and CD86 in mesenteric lymph nodes (MLN). Staining intensities are the same key as for Figure 5; ×10 magnification; inset, ×40, days post SIVmacC8 inoculation. (TIFF) [file pone.0104390.s004.tiff]

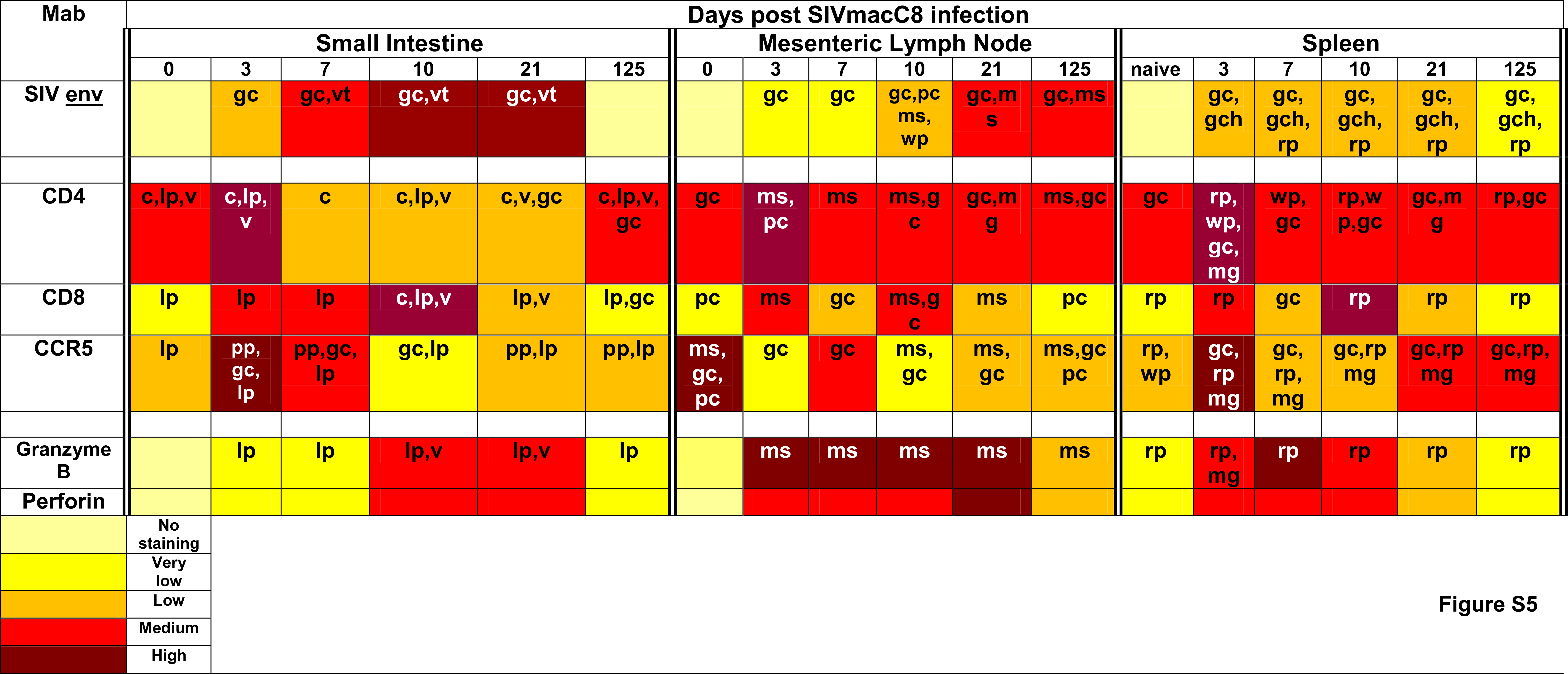

Supplement: Figure S5 — Heatmap showing staining for cell surface markers for viral env, CD4, CD8, CCR5, granzyme B and perforin. Monoclonal antibody staining intensities are shown for SIV env, CD4, CD8, CCR5, granzyme B and perforin. Relative localisation of staining within each tissue is shown: represented by the following key: pp; peyers patches; pc – paracortex, rp – red pulp, 1′ – primary follicle, wp - white pulp, gc – germinal centre, gch, -germinal centre haze, mg – follicular marginal/mantle zone, ms – medullary sinuses, c – crypts, lp – lamina propria, vt – villi tips within each main tissue type. Mab, monoclonal antibody. (TIFF) [file pone.0104390.s005.tiff]

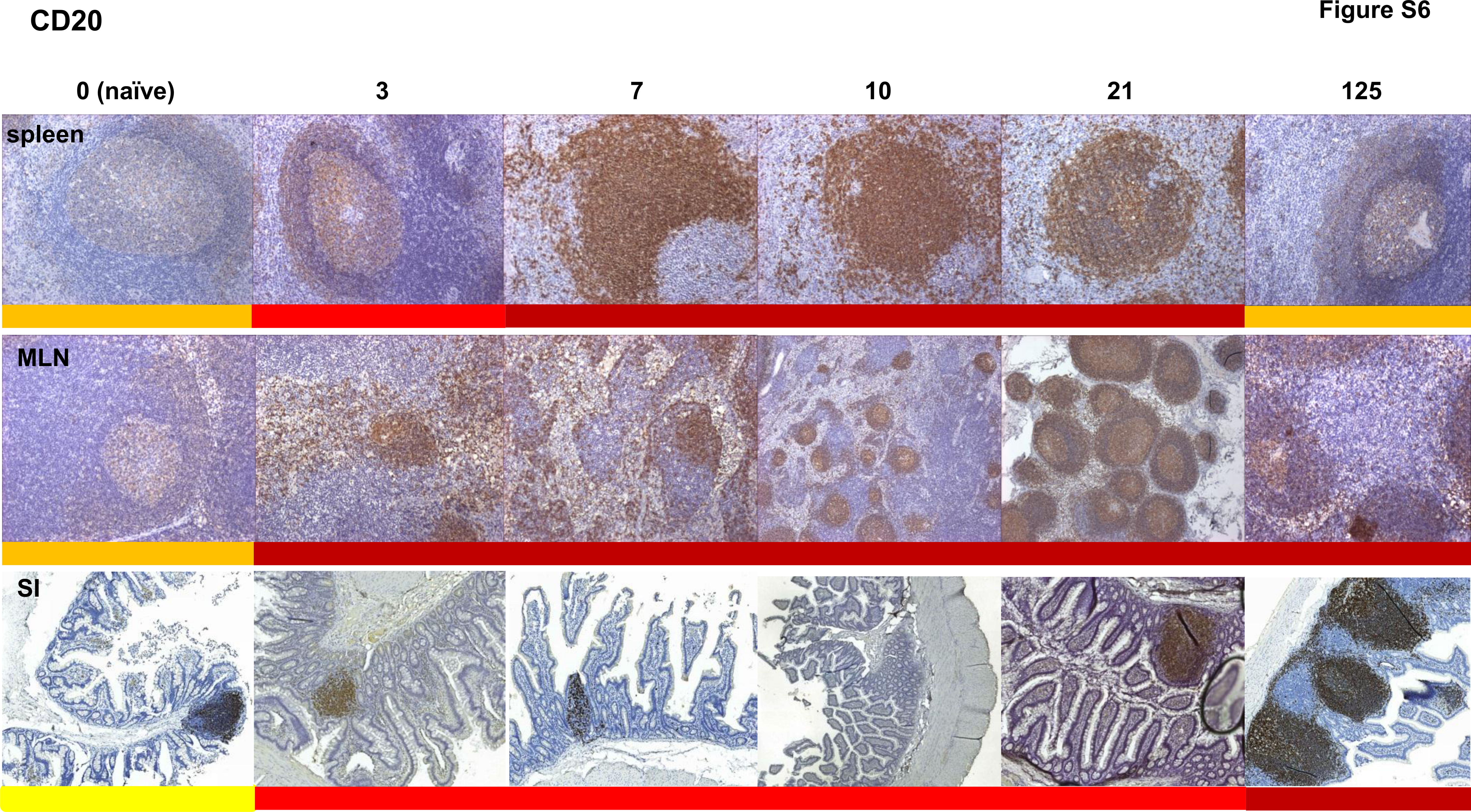

Supplement: Figure S6 — Staining intensities for CD20 B cell marker in spleen, MLN and small intestine during the time course of SIVmacC8 infection. Staining intensities as depicted in Figure 5, days post SIVmacC8 inoculation. (TIFF) [file pone.0104390.s006.tiff]
