# Supplementary material for: Early Biodistribution and Persistence of a Protective Live Attenuated SIV Vaccine Elicits Localised Innate Responses in Multiple Lymphoid Tissues
Source: PLoS One. 2014 Aug 27;9(8):e104390. doi: 10.1371/journal.pone.0104390 (PMC4146474; doi:10.1371/journal.pone.0104390)
Supplement: Table S1 — Quantification of ISH positive cells collected post-mortem for spleen, mesenteric lymph nodes (MLN) and small intestine following SIVmacC8 vaccination (days post-inoculation). (DOCX) [file pone.0104390.s007.docx]

|  | 0 (baseline) | Day 3 | Day 7 | Day 10 | Day 21 | Day 125 |
| --- | --- | --- | --- | --- | --- | --- |
| Spleen | **-** | **+** | **+** | **+++** | **++** | **+** |
| MLN | **-** | **+** | **+** | **+++** | **++** | **+** |
| Small Intestine | **-** | **++** | **++** | **+++** | **+++** | **++** |

**Table S1.** Quantification of ISH positive cells collected *post-mortem* for spleen, mesenteric lymph nodes (MLN) and small intestine following SIVmacC8 vaccination (days post-inoculation). Quantitative data were generated by manually counting all positive cells within 10 random fields of view (x10 lens and x10 eye-piece magnification; equivalent to an area of 2.2mm^2^). The mean number of positive cells/mm^2^ was expressed using a grading system of: + (0.5-6.8), ++ (6.9-13.8) and +++ (>13.8) cells/mm^2^ as indicated.
