# Supplementary material for: Early Biodistribution and Persistence of a Protective Live Attenuated SIV Vaccine Elicits Localised Innate Responses in Multiple Lymphoid Tissues
Source: PLoS One. 2014 Aug 27;9(8):e104390. doi: 10.1371/journal.pone.0104390 (PMC4146474; doi:10.1371/journal.pone.0104390)
Supplement: Table S2 — Comparative immunohistochemistry staining intensities for different markers for dendritic cells, macrophages and B cells. (DOCX) [file pone.0104390.s008.docx]

|  | Days post SIVmacC8 infection (d.p.i) | | | | | | | | | | | | | | | | | |
| --- | --- | --- | --- | --- | --- | --- | --- | --- | --- | --- | --- | --- | --- | --- | --- | --- | --- | --- |
|  | **Small Intestine** | | | | | | **Mesenteric Lymph Node** | | | | | | **Spleen** | | | | | |
|  | **0** | **3** | **7** | **10** | **21** | **125** | **0** | **3** | **7** | **10** | **21** | **125** | **0** | **3** | **7** | **10** | **21** | **125** |
| DC-SIGN | - | - | - | - | - | - | ++ | ++++ | ++++ | ++++ | ++++ | ++++ | ++ | ++++ | ++++ | ++++ | ++++ | ++++ |
| S100 | + | +++ | +++ | +++ | +++ | ++++ | ++ | ++++ | ++++ | ++++ | ++++ | ++++ | ++ | ++++ | ++++ | ++++ | ++++ | ++++ |
| CD40 | + | ++++ | ++++ | ++++ | ++++ | ++++ | +++ | +++ | ++++ | ++++ | ++++ | ++++ | + | +++ | +++ | ++++ | ++++ | ++ |
| CD11c | + | +++ | ++++ | ++++ | ++ | ++ | ++ | ++ | ++ | ++ | +++ | +++ | +++ | +++ | +++ | +++ | ++++ | ++ |
| CD123 | + | +++ | ++++ | ++++ | ++ | ++ | + | ++ | ++ | ++ | +++ | +++ | + | +++ | + | ++++ | ++++ | + |
| CD86 | + | +++ | ++++ | ++++ | ++ | ++ | + | +++ | ++ | ++ | +++ | +++ | + | +++ | +++ | ++++ | ++++ | ++++ |
| CD68 | + | +++ | ++++ | ++++ | ++ | ++ | + | ++ | ++ | +++ | +++ | ++ | + | +++ | +++ | +++ | ++++ | ++++ |
| CD20 | + | +++ | +++ | +++ | +++ | ++++ | ++ | ++++ | ++++ | ++++ | ++++ | ++++ | ++ | +++ | ++++ | ++++ | ++++ | ++ |

**Table S2. Comparative immunohistochemistry staining intensities for different markers for dendritic cells, macrophages and B cells.** A graded system was used to compare each marker for dendritic cells (DC-SIGN, S100, CD40, CD11c, CD123, CD86), macrophages (CD68) and B-cells (CD20). Staining intensities for each antibody are represented as follows: no staining (-), very low (+), low, (++), medium, (+++), and high (++++), matching the colour coding heat-map shown in Figure 5.
