# Supplementary material for: Early Biodistribution and Persistence of a Protective Live Attenuated SIV Vaccine Elicits Localised Innate Responses in Multiple Lymphoid Tissues
Source: PLoS One. 2014 Aug 27;9(8):e104390. doi: 10.1371/journal.pone.0104390 (PMC4146474; doi:10.1371/journal.pone.0104390)
Supplement: Table S3 — Primer sequences for qPCR for multiple gene targets. (DOCX) [file pone.0104390.s009.docx]

| Name | Sequence 5'-3' |
| --- | --- |
|  |  |
| qRPL32-F | CAACATTGGTTATGGGAGCAACA |
| qRPL32-R | TGACGTTGTGGACCAGGAACT |
| qGAPDH-R | GGCTGAGAACGGGAAGCTC |
| qGAPDH-F | AGGGATCTCGCTCCTGGAA |
| qTRIM5α-F | GCGCTACTGGGTTGATGTGACAC |
| qTRIM5α-R | CCCTGGTGCCTGATACATTATCTG |
| qTRIM22-F | ACTCTATTTATGGCTGTGCCTCCC |
| qTRIM22-R | GATGAGTGCTCCATGGTTTGTGAC |
| qA3G-F | TAAACCTTGGGTCAGTGGACAGCA |
| qA3G-R | AAAGGGAATCAGGTCCAGGAAGCA |
| qTHN-F | ACCTGCAACCAGACTGTGATG |
| qTHN-R | CAAGCTCCTCCACTTTCTTTCGTC |
| qIRF7-F | AGAGTCTTCTTCCAAGAGCTGGT |
| qIRF7-R | ACAGCCAGGGCTCCAGCTT |
| qSTAT1-F | CAATACCTCGCACAGTGGTTAGAAAA |
| qSTAT1-R | CGGATGGTGGCAAATGAAAC |
| qSAMHD1-R | TTGTAAGACTGACCCCAACAC |
| qSAMHD1-F | GCGGCATACAAACTCTTTCTG |
|  |  |

**Table S3. Primer sequences for qPCR for multiple gene targets**. RPL-32, GAPDH, TRIM5α, TRIM-22, ApoBEC-3G (A3G), tetherin (THN), IRF-7, STAT-1, SAMHD-1, shown in forward (F) and reverse (R) orientations.
